# Mapping clustered mutations in cancer reveals APOBEC3 mutagenesis of ecDNA

**DOI:** 10.1038/s41586-022-04398-6

**Published:** 2022-02-09

**Authors:** Erik N. Bergstrom, Jens Luebeck, Mia Petljak, Azhar Khandekar, Mark Barnes, Tongwu Zhang, Christopher D. Steele, Nischalan Pillay, Maria Teresa Landi, Vineet Bafna, Paul S. Mischel, Reuben S. Harris, Ludmil B. Alexandrov

**Affiliations:** 1 Department of Cellular and Molecular Medicine, University of California San Diego, La Jolla, CA USA; 2 Department of Bioengineering, University of California San Diego, La Jolla, CA USA; 3 Moores Cancer Center, University of California San Diego, La Jolla, CA USA; 4 Bioinformatics and Systems Biology Graduate Program, University of California San Diego, La Jolla, CA USA; 5 Department of Computer Science and Engineering, University of California San Diego, La Jolla, CA USA; 6 Broad Institute of MIT and Harvard, Cambridge, MA USA; 7 Division of Cancer Epidemiology and Genetics, National Cancer Institute, Bethesda, MD USA; 8 Research Department of Pathology, Cancer Institute, University College London, London, UK; 9 Department of Cellular and Molecular Pathology, Royal National Orthopaedic Hospital NHS Trust, Stanmore, UK; 10 Halıcıoğlu Data Science Institute, University of California San Diego, La Jolla, CA USA; 11 Department of Pathology, Stanford University School of Medicine, Stanford, CA USA; 12 ChEM-H, Stanford University, Stanford, CA USA; 13 Howard Hughes Medical Institute, University of Minnesota, Minneapolis, MN USA; 14 Masonic Cancer Center, University of Minnesota, Minneapolis, MN USA; 15 Institute for Molecular Virology, University of Minnesota, Minneapolis, MN USA; 16 Department of Biochemistry, Molecular Biology and Biophysics, University of Minnesota, Minneapolis, MN USA

**Keywords:** Cancer, Cancer genetics, Cancer genomics

## Abstract

Clustered somatic mutations are common in cancer genomes and previous analyses reveal several types of clustered single-base substitutions, which include doublet- and multi-base substitutions^1–5^, diffuse hypermutation termed omikli^6^, and longer strand-coordinated events termed kataegis^3,7–9^. Here we provide a comprehensive characterization of clustered substitutions and clustered small insertions and deletions (indels) across 2,583 whole-genome-sequenced cancers from 30 types of cancer^10^. Clustered mutations were highly enriched in driver genes and associated with differential gene expression and changes in overall survival. Several distinct mutational processes gave rise to clustered indels, including signatures that were enriched in tobacco smokers and homologous-recombination-deficient cancers. Doublet-base substitutions were caused by at least 12 mutational processes, whereas most multi-base substitutions were generated by either tobacco smoking or exposure to ultraviolet light. Omikli events, which have previously been attributed to APOBEC3 activity^6^, accounted for a large proportion of clustered substitutions; however, only 16.2% of omikli matched APOBEC3 patterns. Kataegis was generated by multiple mutational processes, and 76.1% of all kataegic events exhibited mutational patterns that are associated with the activation-induced deaminase (AID) and APOBEC3 family of deaminases. Co-occurrence of APOBEC3 kataegis and extrachromosomal DNA (ecDNA), termed kyklonas (Greek for cyclone), was found in 31% of samples with ecDNA. Multiple distinct kyklonic events were observed on most mutated ecDNA. ecDNA containing known cancer genes exhibited both positive selection and kyklonic hypermutation. Our results reveal the diversity of clustered mutational processes in human cancer and the role of APOBEC3 in recurrently mutating and fuelling the evolution of ecDNA.

## Abstract (web summary)

An analysis of clustered substitutions and indels across 30 cancer types provides insight into the role of APOBEC3 in giving rise to clustered mutation events through its activity on extrachromosomal DNA.

## Main

Cancer genomes contain somatic mutations that are imprinted by different mutational processes^1,11^. Most single-base substitutions and small indels are independently scattered across the genome; however, a subset of substitutions and indels tend to cluster^12,13^. This clustering has been attributed to a combination of heterogeneous mutation rates across the genome, biophysical characteristics of exogenous carcinogens, dysregulation of endogenous processes and larger mutational events associated with genome instability—amongst others^2,3,6–8,10,13–19^. Previous analyses of clustered mutations have focused on single-base substitutions and revealed several classes of clustered events, including doublet- and multi-base substitutions^1–5^ (DBSs and MBSs, respectively), diffuse hypermutation (omikli)^6^ and longer events (kataegis)^3,7–9^. Most kataegic events were found to be strand-coordinated, defined as sharing the same strand and reference allele^3,11^. Previous studies have also revealed nine clustered signatures^13^ and clustered driver substitutions due to APOBEC3-associated mutagenesis^6^ or carcinogenic-triggered *POLH* mutagenesis^13^.

DBSs have been extensively examined, revealing multiple endogenous and exogenous processes that can cause these events, including failure of DNA repair pathways and exposure to environmental mutagens^1,3,11^. By contrast, MBSs have not been comprehensively investigated, presumably owing to their small numbers in cancer genomes. Moreover, only a handful of processes have been associated with omikli and kataegic events, with most processes attributed to the AID and APOBEC3 family of deaminases^3,6–8,13,14,20–23^. Specifically, the APOBEC3 enzymes, which are typically responsible for antiviral responses^24–30^, give rise to omikli and kataegis by requiring single-stranded DNA as a substrate^6,8,23,31^. Omikli were found to be enriched in early replicating regions and more prevalent in microsatellite stable tumours, indicating that mismatch repair has a role in exposing short single-stranded DNA regions^6^. The differential activity of mismatch repair towards gene-rich regions results in increased omikli events within cancer genes^6^. Kataegis is less prevalent than omikli as it is likely to depend on longer tracks of single-stranded DNA^7,8,19^. Such tracks are typically available during the repair of double-strand breaks and most kataegis has been observed within 10 kb of detected breakpoints^10^.

Amplification of known cancer genes is known to drive tumorigenesis in many types of cancer^32^. Studies have shown high copy-number states of circular ecDNAs, which often contain known cancer genes and are found in most cancers^32–35^. The circular nature of ecDNAs and their rapid replication mimic double-stranded DNA viral pathogens, which indicates that they could be substrates for APOBEC3 mutagenesis; this may contribute to the evolution of tumours that contain ecDNA through accelerated diversification of extrachromosomal oncoproteins.

### The landscape of clustered mutations

**Extended Data Fig. 1:**
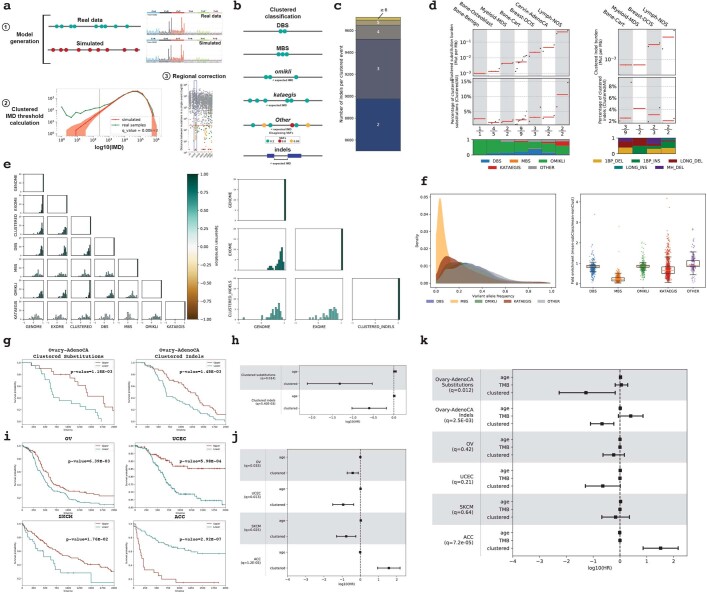
Identification and clinical associations of clustered events. **a**, Schematic depiction for separating clustered mutations for a sample. **b**, Subclassification of clustered substitutions and indels. Expected IMD derived using steps 2 and 3 (**a**). **c**, Distribution of indels present in a single clustered event. **d**, Distribution of clustered substitutions (left) and indels (right) across cancers with less than 10 samples subclassified into different categories. **e**, Correlations between TMB of each sample, the TMB within the exome, or the TMB for each class of clustered substitutions (left) and indels (right). **f**, Distribution of VAFs for all clustered substitution classes (left; DBS: 1,215 samples; MBS: 851; omikli:1,466; kataegis: 1,108; other: 335) with the average fold enrichment compared against non-clustered mutations (right). For each boxplot, the middle line reflects the median, the lower and upper bounds correspond to the first and third quartiles, and the lower and upper whiskers extend from the box by 1.5x the inter-quartile range (IQR). **g**, Kaplan–Meier curves between samples with high (top 80^th^ percentile) and low (bottom 20^th^ percentile) clustered substitution (left) or indel (right) burdens in PCAWG ovarian cancer. **h**, Cox regressions performed for PCAWG cancer types while correcting for age (n = 20 upper and n = 21 lower clustered substitutions; n = 49 upper and n = 49 lower clustered indels). **i**, Kaplan–Meier survival curves for TCGA cancer types with a differential patient outcome associated with the detection of any clustered mutations. **j**, **k**, Cox regressions performed for TCGA samples while correcting for age (**j**) and total mutational burden (**k**) (OV: n = 111 upper, n = 159 lower clustered substitutions; UCEC: n = 322 upper, n = 64 lower; ACC: n = 24 upper, n = 67 lower). PCAWG ovarian cancers were included in **k**. Centre of measure for each Cox regression reflects the log_10_(Hazards ratios) with the 95% confidence intervals in **h**–**k**).

To identify clustered mutations, a sample-dependent intra-mutational distance (IMD) cut-off was derived in which mutations below the cut-off were unlikely to occur by chance (*q*-value < 0.01). A statistical approach using the IMD cut-off, variant allele frequencies (VAFs) and corrections for local sequence context was applied to each specimen (Methods, Extended Data Fig. 1a). Clustered mutations with consistent VAFs were subclassified into four categories (Extended Data Fig. 1b). DBSs and MBSs were characterized as two adjacent mutations (DBSs) and as three or more adjacent mutations (MBSs) (IMD = 1). Multiple substitutions each with IMD > 1 bp and below the sample-dependent cut-off were characterized as either omikli (two to three substitutions) or kataegis (four or more substitutions) (Supplementary Fig. 1). Clustered substitutions with inconsistent VAFs were classified as ‘other’. Although clustered indels were not subclassified into different categories, most events resembled diffuse hypermutation, with 92.3% of events having only two indels (Extended Data Fig. 1c).

**Fig. 1:**
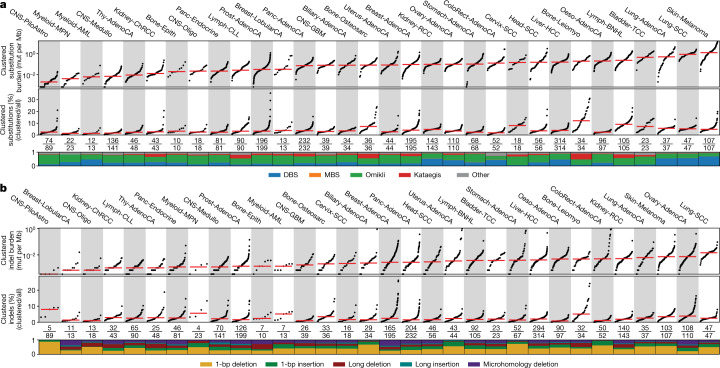
The landscape of clustered mutations across human cancer. **a**, Pan-cancer distribution of clustered substitutions subclassified into DBSs, MBSs, omikli, kataegis and other clustered mutations. Top, each black dot represents a single cancer genome. Red bars reflect the median clustered TMB (mutations (mut) per Mb) for cancer types. Middle, the clustered TMB normalized to the genome-wide TMB reflecting the contribution of clustered mutations to the overall TMB of a given sample. Red bars reflect the median contribution for cancer types. Bottom, the proportion of each subclass of clustered events for a given cancer type with the total number of samples having at least a single clustered event over the total number of samples within a given cancer cohort. **b**, Pan-cancer distribution of clustered small indels. The top and middle panels have the same information as **a**. Bottom, the proportion of each cluster type of indel for a given cancer type with the total number of samples having at least a single clustered indel over the total number of samples within a given cancer cohort. All 2,583 whole-genome-sequenced samples from PCAWG are included in the analysis; however, cancers with fewer than 10 samples were removed from the main figure and included in Extended Data Fig. 1d. For definitions of abbreviations for cancer types used in the figures, see 'Cancer-type abbreviations' in Methods.

Examining 2,583 whole-genome-sequenced cancers from the Pan-Cancer Analysis of Whole Genomes (PCAWG) project revealed a total of 1,686,013 clustered single-base substitutions and 21,368 clustered indels (Fig. 1, Extended Data Fig. 1d). DBSs, MBSs, omikli and kataegis comprised 45.7%, 0.7%, 37.2% and 7.0% of clustered substitutions across all samples, respectively, and their distributions varied greatly within and across cancer types. For example, melanoma had the highest clustered substitution burden, with ultraviolet light associated doublets (CC>TT) accounting for 74.2% of clustered mutations; however, these contributed only 5.3% of all substitutions in melanoma (Fig. 1a). By contrast, 11.5% of all substitutions in bone leiomyosarcomas were clustered, and omikli and kataegis constituted 43.8% and 46.7% of these mutations, respectively (Fig. 1a). Clustered indels exhibited similarly diverse patterns within and across cancer types (Fig. 1b). For example, the highest mutational burden of clustered indels was observed in lung and ovarian cancers. Clustered indels in lung cancer accounted for only 2.6% of all indels and were characterized by 1-bp deletions. By contrast, clustered long indels at microhomologies were commonly found in ovarian and breast cancers and contributed more than 10% of all indels in a subset of samples (Fig. 1b). Correlations between the total number of mutations and the number of clustered mutations were observed for DBSs and omikli but not for MBSs, kataegis or indels (Extended Data Fig. 1e). In most cancers, DBSs and omikli had VAFs consistent with those of non-clustered mutations, whereas MBSs and kataegis tended to have lower VAFs (Extended Data Fig. 1f). Kataegic events contained 4 to 44 mutations and 81% of events were strand-coordinated, indicative of damage or enzymatic changes on a single DNA strand.

The overall survival was compared between patients with cancers containing high and low numbers of clustered mutations within whole-genome-sequenced PCAWG and whole-exome sequenced The Cancer Genome Atlas (TCGA) cancer types^36^. Better overall survival was observed only in whole-genome-sequenced ovarian cancers that contained high-levels of clustered substitutions or clustered indels (*q*-values < 0.05) (Extended Data Fig. 1g, h). Conversely, whole-exome-sequenced adrenocortical carcinomas containing clustered substitutions were associated with a worse overall survival (*q*-value = 7.2 × 10^−5^) (Extended Data Fig. 1i–k).

### Signatures of clustered mutations

**Fig. 2:**
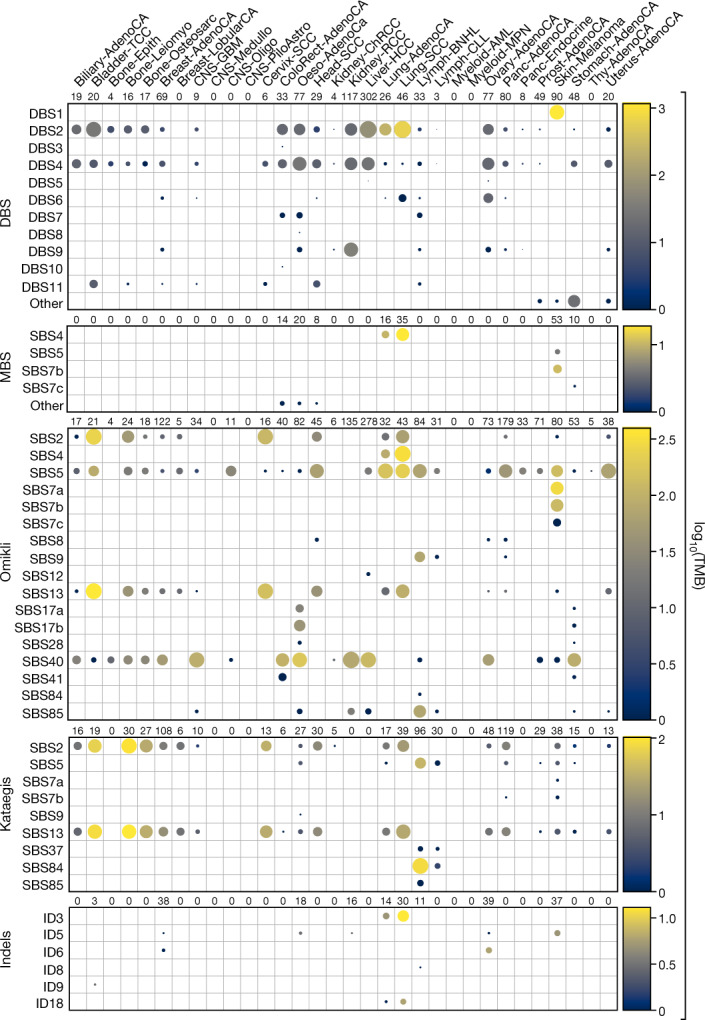
Mutational processes that underlie clustered events. Each circle represents the activity of a signature for a given cancer type. The radius of the circle determines the proportion of samples with greater than a given number of mutations specific to each subclass; the colour reflects the median number of mutations per cancer type. A minimum of two samples are required per cancer type for visualization (Methods).

**Extended Data Fig. 2:**
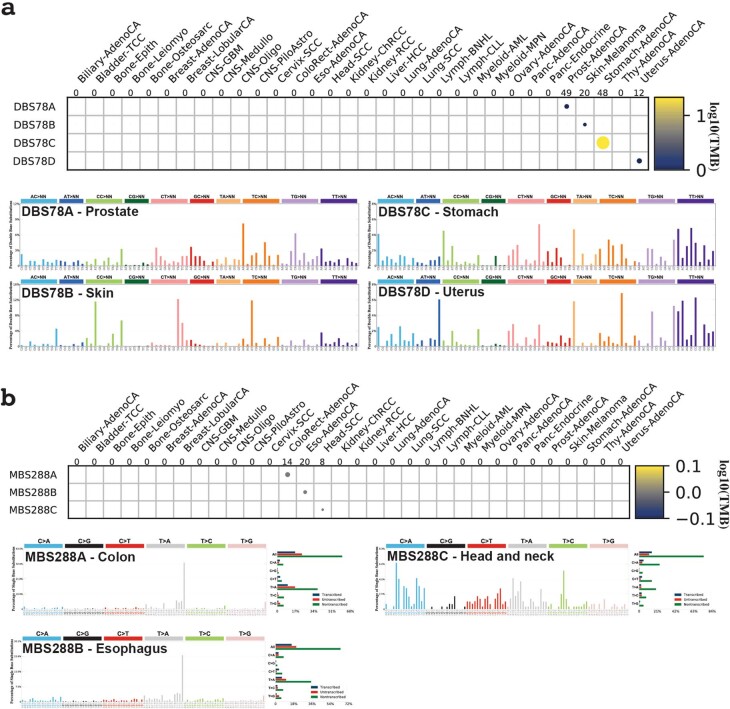
De novo signatures of DBS and MBS signatures. **a**, The activity of DBS de novo signatures (top) and the corresponding signatures extracted from prostate, skin, stomach, and uterine cancers that could not be accurately reconstructed using known COSMIC mutational signatures (bottom; Methods). **b**, The activity of MBS de novo signatures (top) and the corresponding signatures extracted from colon, oesophagus, and head and neck cancers that could not be accurately reconstructed using known COSMIC mutational signatures (bottom; Methods).

Mutational signature analysis was performed for each category of clustered events, which enabled the identification of 12 DBS, 5 MBS, 17 omikli, 9 kataegic and 6 clustered indel signatures (Fig. 2, Supplementary Tables 1–5). Although DBS signatures have previously been described^1^, previous analysis combined DBSs and MBSs into a single class^1^. Separating these events into individual classes showed that a multitude of processes can give rise to DBSs, whereas most MBSs are attributable to signatures associated with tobacco smoking (SBS4) or ultraviolet light (SBS7). Additional DBS and MBS signatures were found within a small subset of cancer types (Extended Data Fig. 2).

**Extended Data Fig. 3:**
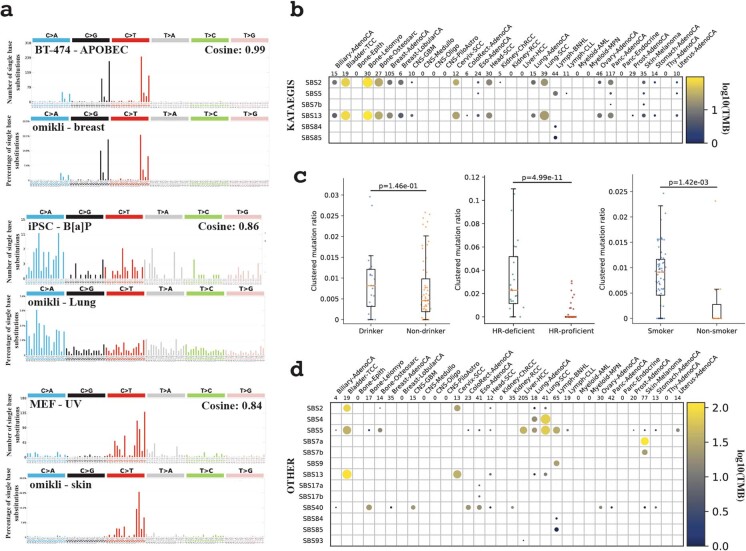
Experimental validation and epidemiological associations of clustered mutational processes. **a**, Experimental validation of three omikli processes. Specifically, APOBEC3-associated omikli were validated using a clonally expanded BT-474 breast cancer cell line (top), omikli events resulting from exposure to benzo[*a*]pyrene were validated using iPS cells (middle), and omikli events resulting from exposure to ultraviolet light were validated using iPS cells (bottom). **b**, Mutational processes of strand-coordinated kataegic events. **c**, Epidemiological associations comparing the ratio of clustered TMB to the total TMB for a given sample between: drinkers (n = 25) and non-drinkers (n = 61); smokers (n = 68) and non-smokers (n = 11); homologous-recombination deficient (HR-deficient; n = 25) and homologous-recombination proficient samples (HR-proficient; n = 64). For each boxplot, the middle line reflects the median, the lower and upper bounds of the box correspond to the first and third quartiles, and the lower and upper whiskers extend from the box by 1.5x the inter-quartile range (IQR). P-values were calculated using a two-tailed Mann–Whitney *U*-test. **d**, Mutational processes of clustered events with inconsistent VAFs classified as other clustered substitutions. A minimum of two samples are required per cancer type for visualization (Methods).

In cancer genomes, omikli were previously attributed to APOBEC3 mutagenesis^6^ with some indirect evidence from experimental models^23,37,38^. Our analysis of sequencing data^39^ from the clonally expanded breast cancer cell line BT-474 with active APOBEC3 mutagenesis experimentally confirmed the existence of APOBEC3-associated omikli events (cosine similarity: 0.99) (Extended Data Fig. 3a). Only 16.2% of omikli events across the 2,583 cancer genomes matched the APOBEC3 mutational pattern, suggesting that a variety of other processes can give rise to diffuse clustered hypermutation. Notably, our analysis revealed omikli due to tobacco smoking (SBS4), clock-like mutational processes (SBS5), ultraviolet light (SBS7), both direct and indirect mutations from AID (SBS9 and SBS85), and multiple mutational signatures with unknown aetiology in different cancer types (SBS8, SBS12, SBS17a/b, SBS28, SBS40 and SBS41) (Fig. 2). Cell lines previously exposed to benzo[*a*]pyrene^40^ and ultraviolet light^41^ confirmed the generation of omikli events as a result of these two environmental exposures (cosine similarities: 0.86 and 0.84, respectively) (Extended Data Fig. 3a).

**Extended Data Fig. 4:**
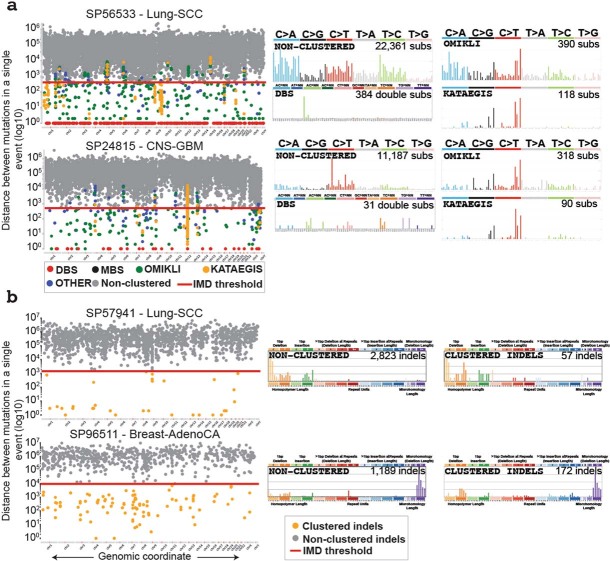
Examples of clustered mutational signatures. **a**, Two samples depicting the intra-mutational distance (IMD) distributions of substitutions across genomic coordinates, where each dot represents the minimum distance to adjacent mutations for a selected mutation coloured based on the corresponding subclassification of event (rainfall plot; left). The red lines depict the sample-dependent IMD threshold for each sample. Specific clustered mutations may be above this threshold based on corrections for regional mutation density. The mutational spectra for the different catalogues of clustered and non-clustered substitutions for each sample (right; MBS are not shown). **b**, Two samples illustrating the IMD distributions of indels across the given genomes, with the IMD indel thresholds shown in red (left). The non-clustered and clustered indel catalogues for each sample (right).

Of the nine kataegic signatures, four have been reported previously, including two associated with APOBEC3 deaminases (SBS2 and SBS13) and two associated with canonical or non-canonical AID activities (SBS84 and SBS85) (Fig. 2). SBS5 (clock-like mutagenesis) accounted for 15.0% of kataegis, with most events occurring in the vicinity of AID kataegis within B cell lymphomas. The remaining four kataegic signatures accounted for only 8.9% of kataegic mutations and included SBS7a/b (ultraviolet light), SBS9 (indirect mutations from AID) and SBS37 (unknown aetiology). Most kataegic signatures were strand-coordinated (Extended Data Fig. 3b). Some samples exhibited consistent whereas others exhibited distinct signatures of clustered and non-clustered mutagenesis (Extended Data Fig. 4). For example, in SP56533 (lung squamous cell carcinoma), most non-clustered and omikli substitutions were caused by tobacco signature SBS4, whereas kataegic events were generated by the APOBEC3 signatures (Extended Data Fig. 4a). By contrast, the pattern of non-clustered substitutions in SP24815 (glioblastoma) was due to clock-like signatures SBS1 and SBS5, whereas omikli and kataegic events were mostly attributable to APOBEC3 (Extended Data Fig. 4a).

The remaining ‘other’ clustered substitutions exhibited inconsistent VAFs that probably represent mutations at highly mutable genomic regions or the effects of co-occurring large mutational events such as copy number alterations (Extended Data Fig. 3d, Supplementary Table 6).

Different cancers showed distinct tendencies of clustered indel mutagenesis (Fig. 2). For instance, clustered indels attributed to ID3 (tobacco smoking; characterized by 1-bp deletions) were found predominately in lung cancers and were significantly increased in smokers compared to non-smokers (*P* = 0.0014) (Extended Data Figs. 3c, 4b). Clustered indels due to signatures ID6 and ID8—both attributed to homologous recombination deficiency and characterized by long indels at microhomologies—were found in breast and ovarian cancers and were highly increased in cancers with known deficiencies in homologous recombination genes (*P* = 4.9 × 10^−11^) (Extended Data Figs. 3c, 4b).

### Panorama of clustered driver mutations

**Fig. 3:**
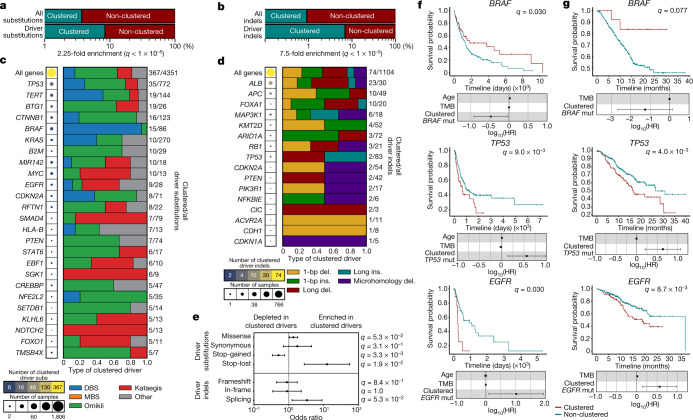
Panorama of clustered driver mutations in human cancer. **a**, **b**, Percentage of clustered mutations (top) compared to the percentage of clustered driver events (bottom) for substitutions (**a**) and indels (**b**). **c**, The frequency of clustered driver events across known cancer genes. The radius of the circle is proportional to the number of samples with a clustered driver mutation within a gene; the colour reflects the clustered mutational burden. All clustered driver events are classified into one of the five clustered classes, with the number of clustered driver substitutions and the total number of driver substitutions shown on the right. **d**, Clustered indel drivers are shown in a similar manner to **c**. **e**, The odds ratio of clustered substitutions (top) and indels (bottom) resulting in deleterious (*n* = 192 clustered substitutions; *n* = 54 clustered indels) or synonymous changes (*n* = 5 clustered substitutions; *n* = 5 clustered indels) within a given driver gene compared to non-clustered driver mutations (*n* = 771 deleterious and *n* = 237 synonymous substitutions; *n* = 111 deleterious and *n* = 50 synonymous indels). All events were overlapped with the PCAWG consensus list of driver events and were annotated using the ENSEMBL Variant Effect Predictor (VEP). The odds ratios are shown with their 95% confidence intervals. **f**, Kaplan–Meier survival curves comparing the outcome of samples with clustered versus non-clustered mutations in *BRAF* (top), *TP53* (middle) and *EGFR* (bottom) across TCGA cohorts. Only cohorts with more than five samples containing a clustered mutation within the given gene were included. **g**, Kaplan–Meier survival curves comparing the outcome of samples with clustered versus non-clustered mutations in the same genes across the MSK-IMPACT cohort. The log_10_-transformed hazards ratios (log_10_(HR)) are shown with their 95% confidence intervals in **f**, **g**. Cox regressions were corrected for age (TCGA only), mutational burden and cancer type (Methods). *Q* values in **a**, **b**, **e** were calculated using a two-tailed Fisher’s exact test and corrected for multiple hypothesis testing.

**Extended Data Fig. 5:**
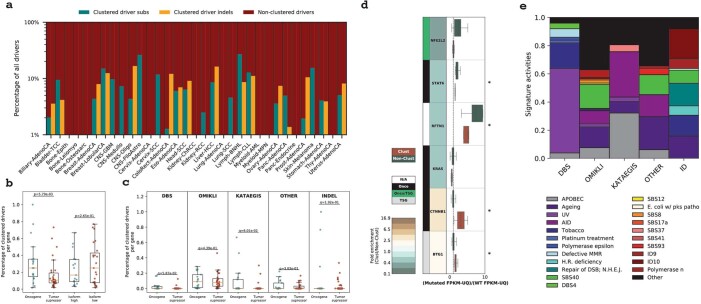
Mutational processes of clustered driver events. **a**, The percentage of clustered driver substitutions and indels within each cancer type. All samples 2,583 whole-genome sequenced samples from PCAWG with a detected driver event are included; however, cancer types with fewer than 10 samples are not presented. **b**, The proportion of clustered driver mutations per cancer gene compared between oncogenes (n = 19 genes) versus tumour suppressor genes (n = 30 genes) and genes with high numbers of isoforms (n = 17) versus genes with low numbers of isoforms (n = 23; upper and lower quartiles of isoforms across all cancer drivers). **c**, The proportion of clustered driver mutations for a given subclass per cancer gene compared between oncogenes (n = 17 genes with clustered substitutions and n = 13 with for clustered indels) versus tumour suppressor genes (n = 28 genes with clustered substitutions and n = 70 genes with clustered indels). **d**, The relative expression of driver genes containing clustered (copper) versus non-clustered events (green). All expression values were normalized using FPKM normalization and upper quartile normalization obtained from the official PCAWG release and were subsequently normalized using the average expression of the wild-type gene. A value of 1 (dashed lined) reflects no difference in expression compared to the wild-type gene. **e**, The proportional activity of mutational signatures contributing to clustered driver events within each subclass. MBSs did not contribute to any reported driver events. For analyses in **b**–**d**, p-values were generated using a two-tailed Mann–Whitney *U*-test (*P < 0.05; p = 0.03 for *STAT6*; p = 0.04 for *CTNNB1*; p = 0.02 for *BTG1*). For each boxplot, the middle line reflects the median, the lower and upper bounds of the box correspond to the first and third quartiles, and the lower and upper whiskers extend from the box by 1.5x the inter-quartile range (IQR).

The PCAWG project elucidated a constellation of mutations that putatively drive cancer development^10^. Our current analysis reveals significant enrichments of clustered substitutions and clustered indels amongst these driver mutations. Specifically, whereas only 3.7% of all substitutions and 0.9% of all indels are clustered events, they contribute 8.4% and 6.9% of substitution and indel drivers, respectively (*q*-values < 1 × 10^−5^; Fisher’s exact tests) (Fig. 3a, b). Omikli accounted for 50.5% of all clustered substitution drivers, whereas DBSs, kataegis and other clustered events each contributed between 14% and 18% (Fig. 3c). Clustered driver substitutions varied greatly between genes and across different cancers (Fig. 3c, Extended Data Fig. 5a) with a 2.4-fold enrichment of clustered events within oncogenes compared to tumour suppressors (*P* = 5.79 × 10^−3^) (Extended Data Fig. 5b, c). In some cancer genes, only a small percentage of driver events are due to clustered substitutions; examples include *TP53* (4.5% clustered driver substitutions), *KRAS* (3.7%) and *PIK3CA* (2.2%). In other genes, most detected substitution drivers were clustered events; examples include: *BTG1* (73.1%), *SGK1* (66.6%), *EBF1* (60.0%) and *NOTCH2* (38.5%). Notably, the contribution from each class of clustered events varied across driver substitutions in different genes (Fig. 3c). For instance, ultraviolet-light-associated DBSs comprised 93% of clustered *BRAF* driver events, omikli contributed 63% of clustered *BTG1* driver events and kataegis accounted for 100% of clustered *NOTCH2* driver substitutions (Fig. 3c). Similar behaviour was observed for clustered indel drivers, with 48.7% being single-base pair indels (Fig. 3d). In some cancer genes, clustered indel drivers were rare (for example, 2.4% of indel drivers in *TP53* were clustered), whereas in others they were common (for example, 76.6% in *ALB*) (Fig. 3d). Clustered driver substitutions were enriched in stop-lost mutations (*q*-value = 1.9 × 10^−2^) and depleted in stop-gained mutations (*q*-value = 3.3 × 10^−3^) when compared to non-clustered drivers (Fig. 3e). Furthermore, driver genes that contained clustered events were often differentially expressed compared to those containing non-clustered events (Extended Data Fig. 5d). For instance, clustered events within *CTNNB1* and *BTG1* associated with an increased expression compared to both non-clustered and wild-type expression levels for each gene (*q*-values < 0.05). Opposite effects were observed in *STAT6* and *RFTN1* (*q*-values < 0.05). Collectively, these driver events were induced by the activity of multiple mutational processes including exposure to ultraviolet light, tobacco smoke, platinum chemotherapy and AID and APOBEC3 activity; amongst others (Extended Data Fig. 5e).

The clinical utility of detecting clustered events in driver genes was evaluated by comparing the survival amongst individuals with clustered mutations versus individuals with non-clustered mutations within each driver gene across all whole-exome-sequenced samples in TCGA. For each of these comparisons, we performed Cox regressions considering the effects from age and tumour mutational burden (TMB) while correcting for cancer type and multiple hypothesis testing. These results were validated in targeted panel sequencing data from the Memorial Sloan Kettering-Integrated Mutation Profiling of Actionable Cancer Targets (MSK-IMPACT) cohort^42,43^. These analyses revealed a significant difference in survival between individuals with clustered and individuals with non-clustered mutations detected in *TP53*, *EGFR* and *BRAF*. Specifically, individuals with clustered events within *BRAF* had a better overall survival compared to individuals with non-clustered events (*q*-values < 0.05) (Fig. 3f, g). Conversely, in both TCGA and MSK-IMPACT, individuals with clustered mutations in *TP53* or *EGFR* exhibited a significantly worse outcome compared to individuals with non-clustered mutations in each of these genes (*q*-values < 0.05) (Fig. 3f, g).

### Kataegic events and focal amplifications

**Fig. 4:**
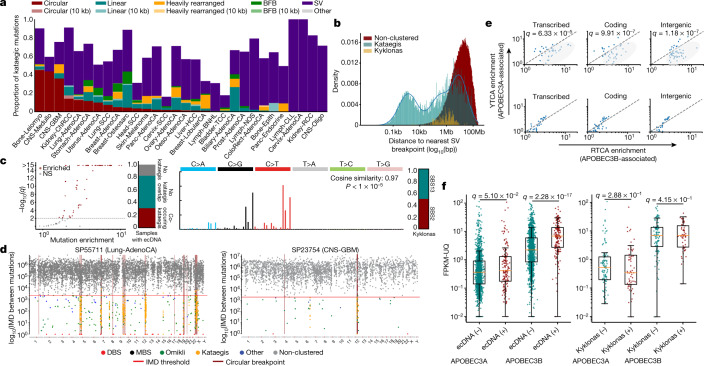
Kataegic events co-locate with most forms of structural variation. **a**, Proportion of all kataegic events per cancer type overlapping different amplifications or structural variations. **b**, Distance to the nearest breakpoint for all kataegic mutations (teal), kyklonas (gold) and non-clustered mutations (red). Kataegic distances were modelled as a Gaussian mixture with three components (blue line). **c**, Left, volcano plot depicting samples that are statistically enriched for kyklonas (red; *q*-values from a false discovery rate (FDR)-corrected *z*-test; not significant (NS)). Middle left, proportion of samples with ecDNA co-occurring with kataegis. Middle right, mutational spectrum of all kyklonas. Right, proportion of kyklonic events attributed to SBS2 and SBS13. Cosine similarity was calculated between the kyklonic and the reconstructed spectra composed using SBS2 and SBS13 (*P* value from a *Z*-score test). **d**, Rainfall plots illustrating the IMD distribution for a given sample with the genomic locations of ecDNA breakpoints (maroon). **e**, Top, YTCA versus RTCA enrichments per sample with kyklonas, in which YTCA or RTCA enrichment is suggestive of higher APOBEC3A or APOBEC3B activity, respectively. Genic mutations were divided into transcribed (template strand) and coding mutations. The RTCA/YTCA fold enrichments were compared to those of non-clustered mutations (bottom). **f**, Relative expression of APOBEC3A and APOBEC3B in samples containing ecDNA (*n* = 157) compared to samples without ecDNA (*n* = 1,364) (left), and in samples with ecDNA that have kyklonas (*n* = 59) compared to samples without kyklonas (*n* = 98) (right). Expression values were normalized using fragments per kilobase of exon per million mapped fragment (FPKM) and upper quartile (UQ) normalization obtained from the PCAWG release. *Q* values in **e**, **f** were calculated using a two-tailed Mann–Whitney *U*-test and FDR corrected using the Benjamini–Hochberg procedure. For box plots, the middle line reflects the median, the lower and upper bounds of the box correspond to the first and third quartiles, and the lower and upper whiskers extend from the box by 1.5× the interquartile range.

**Extended Data Fig. 6:**
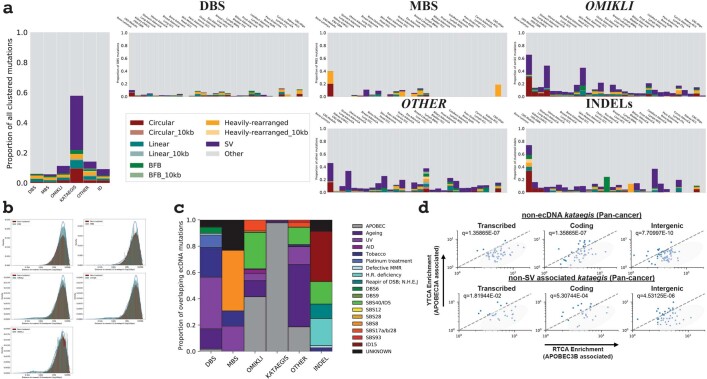
Clustered events and structural variations. **a**, The proportion of all clustered events co-locating with structural variations across all cancer types (left) and across each cancer type (right). **b**, The distance to the nearest structural variation for each class of clustered mutations (teal), and non-clustered mutations (red). The distribution for each class of clustered events were modelled using a Gaussian mixture (blue line). DBSs and MBSs were modelled using a single distribution, whereas omikli, other, and indels were modelled using two components reflecting the minimal distribution of overlap with structural variations. **c**, The mutational signatures active in ecDNA clustered events. **d**, YTCA versus RTCA enrichments per sample within non-ecDNA kataegis (top) and non-SV associated kataegis (bottom), where YTCA and RTCA enrichment is suggestive of APOBEC3A or APOBEC3B activity, respectively. Genic mutations were divided into transcribed (template strand) and coding mutations. The RTCA/YTCA fold enrichments were compared to the fold enrichments of non-clustered mutations (p-values calculated using two-tailed Mann–Whitney *U*-tests and corrected for multiple hypothesis testing using the Benjamini–Hochberg FDR procedure).

In each sample, kataegic mutations were separated into distinct events on the basis of consistent VAFs across adjacent mutations and IMD distances greater than the sample-dependent IMD threshold (Methods). Our analysis revealed that 36.2% of all kataegic events occurred within 10 kb of a structural breakpoint but not on detected focal amplifications (Fig. 4a). In addition, 21.8% of all kataegic events occurred either on a detected focal amplification or within 10 kb of a focal amplification’s structural breakpoints: 9.6% on circular ecDNA, 6.3% on linear rearrangements, 3.3% within heavily rearranged events and 2.6% associated with breakage–fusion–bridge cycles (BFBs) (Fig. 4a). Finally, 42.0% of kataegic events were neither within 10 kb of a structural breakpoint nor on a detected focal amplification. Modelling the distribution of the distances between kataegic events and the nearest structural variations revealed a multi-modal distribution with three components (Fig. 4b): kataegis within 10 kb, around 1 Mb, or more than 1.5 Mb of a detected breakpoint. Of note, ecDNA-associated kataegis—termed kyklonas (Greek for cyclone)—had an average distance from the nearest breakpoint of around 750 kb, with only 0.35% of kyklonic events occurring both on ecDNA and within 10 kb of a breakpoint (Fig. 4b). These results indicate that kyklonic events are not likely to have occurred because of structural rearrangements during the formation of ecDNA. In most cancer types, DBSs, MBSs, omikli and other cluster events were not found in the vicinity of structural variations (Extended Data Fig. 6a, b).

### Recurrent kyklonic mutagenesis of ecDNA

Although only 9.6% of kataegic events occur within ecDNA regions, more than 30% of ecDNAs had one or more associated kyklonic events (Fig. 4c). The mutations within these ecDNA regions were dominated by the APOBEC3 patterns, which are characterized by strand-coordinated C>G and C>T mutations in the TpCpW context and attributed to signatures SBS2 and SBS13 (*P* <1 × 10^−5^) (Fig 4c, d, Extended Data Fig. 6c). These APOBEC3-associated events contributed 97.8% of all kyklonic events, whereas the remaining mutations were attributed to clock-like signature SBS5 (1.2%) and other signatures (1.0%) (Extended Data Fig. 6c). Furthermore, kyklonic events exhibited an enrichment of C>T and C>G mutations at APOBEC3B-preferred RTCA compared to APOBEC3A-preferred YTCA contexts (underlining reflects the mutated nucleotide)^7^, indicating that APOBEC3B is likely to have an important role in the mutagenesis of circular DNA bodies (Fig. 4e). Similar levels of enrichment for RTCA contexts were also observed in both non-ecDNA kataegis and non-structural variant (SV)-associated kataegis, suggesting that APOBEC3B generally gives rise to many of the strand-coordinated kataegic events (Extended Data Fig. 6d). An increase in the expression of APOBEC3B—but not APOBEC3A—was observed in cancers with ecDNA compared to samples without ecDNA (3.1-fold; *q*-value < 1 × 10^−5^) (Fig. 4f). Within cancers containing ecDNA, no differences were observed in the expression of APOBEC3A or APOBEC3B between samples with and without kyklonic events (Fig. 4f).

**Fig. 5:**
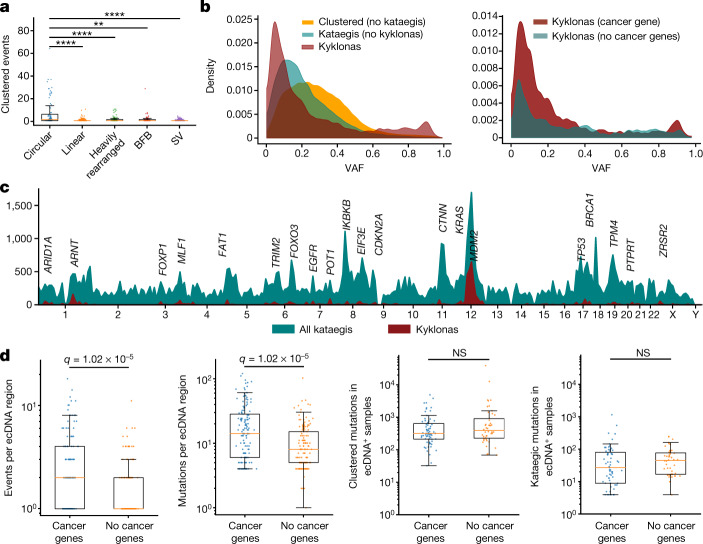
Recurrent APOBEC3 hypermutation of ecDNA. **a**, Number of clustered events overlapping a single amplicon or SV event; each dot represents an amplicon or SV (*n* = 84 circular; *n* = 275 linear; *n* = 111 heavily rearranged; *n* = 62 BFB; and *n* = 11,139 SV). A 10-kb window was used to determine the co-occurrence of kataegis with SV breakpoints (**q < 0.01, ****q < 0.0001). **b**, Left, normalized distributions of the VAFs for all clustered mutations excluding kataegis (orange), all non-ecDNA kataegis (teal), and kyklonas (red). Right, normalized VAF distributions for kyklonic ecDNA containing cancer genes and for kyklonic ecDNA without cancer genes. **c**, Frequency of recurrence for all kataegis (teal) and kyklonas (red) using a sliding genomic window of 10 Mb. **d**, Number of kyklonic events and kyklonic mutations per ecDNA region containing cancer genes (*n* = 137) or without cancer genes (*n* = 134; left and right, respectively). **e**, Total number of clustered and kataegic mutations found in samples with ecDNAs containing cancer genes (*n* = 67 samples) compared to samples with ecDNAs without cancer genes (*n* = 44; left and right, respectively). *Q* values in **a**, **d**, **e** were calculated using a two-tailed Mann–Whitney *U*-test and FDR-corrected using the Benjamini–Hochberg procedure. Box plot parameters as in Fig. 4.

**Extended Data Fig. 7:**
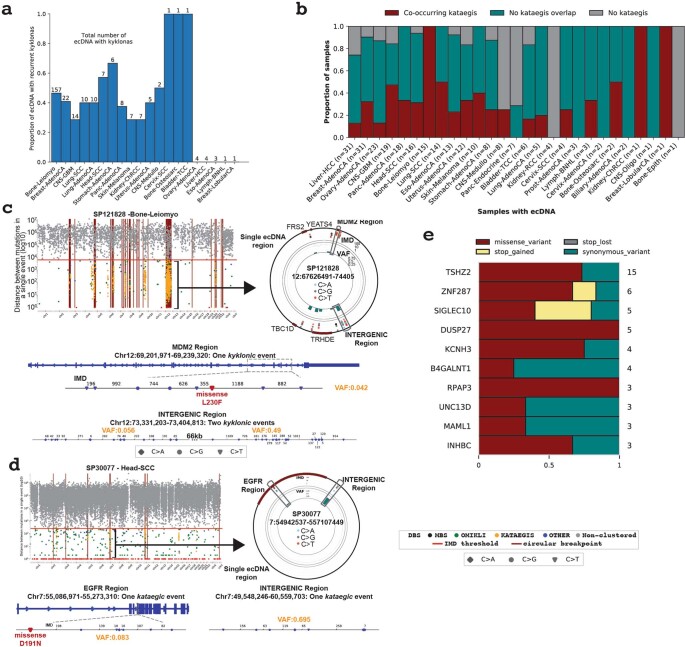
Recurrent mutagenesis and functional effects of kyklonas. **a**, The total number of recurrently mutated ecDNA displayed as a proportion of the total number of ecDNA with kyklonas for a given cancer type. The total number of ecDNA with kyklonas are displayed above each bar plot for each cancer type. All ecDNA with recurrent hypermutation were considered enriched for kyklonic events after correcting for multiple hypothesis testing (Z-score test; q-values < 0.05). **b**, Proportion of samples containing ecDNA divided exclusively into those with co-occurring kataegis, no kataegis overlap, and no detected kataegis across the entire genome. The number of samples included in each cancer type are listed. For certain cancer types, as few as a single sample may represent the entire proportional breakdown (for example, Bone-Osteosarc or Bone-Epith). **c**, A single sarcoma genome and **d**, a single head squamous cell carcinoma genome depicting the overlap of kataegis with ecDNA regions displayed as a rainfall (top left) with a single zoomed in ecDNA represented using a circos plot (top right). Bottom: Two regions of the ecDNA with overlapping kyklonic events. VAFs are shown per event (orange). **e**, Kyklonic substitutions resulting in recurrent coding mutations within known cancer genes.

More recurrent APOBEC3 kataegis was observed across circular ecDNA regions compared to other forms of structural variation (Fig. 5a). An average of 2.5 kyklonic events were observed within ecDNA regions (range: 0–64 kyklonic events; 0–505 mutations). Recurrent kyklonas was widespread across cancer types (Extended Data Fig. 7a, b). For example, glioblastomas and sarcomas exhibited an average of 5 and 86 kyklonic mutations, respectively. The average VAF of kyklonas was significantly lower than both non-ecDNA associated kataegis and all other clustered events (*q*-values < 1 × 10^−5^ Fig. 5b). Notably, a subset of kyklonas exhibited VAFs above 0.80, which is likely to reflect early mutagenesis of genomic regions that have subsequently amplified as ecDNA. Moreover, kyklonic events with high VAFs occurred more commonly on ecDNA that contained known cancer genes, suggesting a mechanism of positive selection (Fig. 5b). Approximately 7.2% of kyklonas occurred early in the evolution of a given ecDNA population within a tumour (VAF > 0.80), whereas the majority of kyklonic events (around 82.5%; VAF < 0.5) have probably occurred after clonal amplification by recurrent APOBEC3 mutagenesis.

Recurrent kyklonic events were increased within or near known cancer-associated genes including *TP53*, *CDK4* and *MDM2*, amongst others (Fig. 5c). These recurrent kyklonas were observed across many cancers including glioblastomas, sarcomas, head and neck carcinomas and lung adenocarcinomas (Extended Data Fig. 7c, d). For example, in a sarcoma sample (SP121828), 10 distinct kyklonic events overlapped a single ecDNA region with recurrent APOBEC3 activity in proximity to *MDM2*, resulting in a missense L230F mutation (Extended Data Fig. 7c). The same ecDNA region contained additional kyklonic events occurring within intergenic regions that have distinguishable VAF distributions, implicating recurrent mutagenesis (Extended Data Fig. 7c). Similarly, two distinct kyklonic events occurred on an ecDNA containing *EGFR*, resulting in a missense mutation D191N within a head and neck cancer (Extended Data Fig. 7d). Of note, ecDNA regions with known cancer-associated genes had significantly higher numbers of kyklonic events and mutational burdens of kyklonas compared to ecDNA regions without any known cancer-associated genes (*q*-values < 1 × 10^−5^) (Fig. 5d). Furthermore, we observed a higher co-occurrence of kyklonas with known cancer-associated genes, which were mutated 2.5 times more than ecDNA without cancer-associated genes (*P* = 1.2 × 10^−5^; Fisher’s exact test). Overall, 41% of kyklonic events were found within the footprints of known cancer driver genes (*P* < 1 × 10^−5^). These enrichments cannot be accounted for either by an increase in the overall mutations or by an increase in the overall clustered mutations in these samples (Fig. 5e). To understand the functional effect of kyklonas, we annotated the predicted consequence of each mutation. In total, 2,247 kyklonic mutations overlapped putative cancer-associated genes, of which 4.3% occur within coding regions (Extended Data Fig. 7e). Specifically, 63 resulted in missense mutations, 29 resulted in synonymous mutations, 4 introduced premature stop codons and 1 removed a stop codon (Supplementary Table 7). These downstream consequences of APOBEC3 mutagenesis suggest a contribution to the oncogenic evolution of specific ecDNA populations.

### Validation of kyklonic events in ecDNA

**Extended Data Fig. 8:**
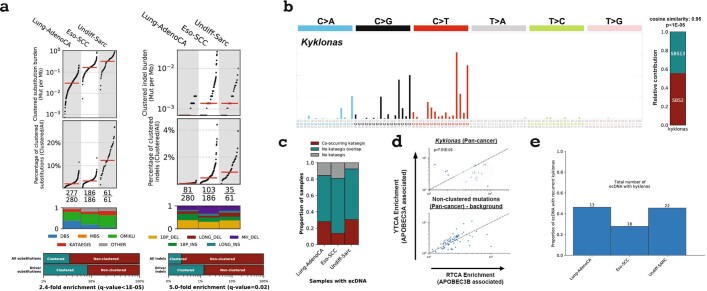
Validation of APOBEC3 hypermutation of ecDNA in three independent cohorts. **a**, Distribution of clustered substitutions (left) and clustered indels (right) across three validation cohorts. Clustered substitutions were subclassified into DBSs, MBSs, omikli, kataegis, and other clustered mutations. Top: Each black dot represents a single cancer genome. Red bars reflect the median clustered TMB and the percentage of clustered mutations contributing to the overall TMB of a given sample for each cancer type. Middle: The proportion of each subclass of clustered events for a given cancer type with the total number of samples having at least a single clustered event over the total number of samples within a given cancer cohort. Bottom: Percentage of clustered mutations compared to the percentage of clustered driver events for substitutions (left) and indels (right). P-values were calculated using a Fisher’s exact test and corrected for multiple hypothesis testing using Benjamini–Hochberg FDR procedure. **b**, Left: The mutational spectrum of all kyklonas across the validation cohorts. Right: The proportion of kyklonic events attributed to SBS2 and SBS13 (p-value determined using a Z-score test; Methods). **c**, The proportion of samples with ecDNA that co-occur with kataegis, do not co-occur with kataegis, or do not have any detected kataegic activity across each cohort. **d**, YTCA versus RTCA enrichments per sample with kyklonas, where YTCA and RTCA enrichment is suggestive of higher APOBEC3A or APOBEC3B activity, respectively. The RTCA/YTCA fold enrichments were compared to the fold enrichments of non-clustered mutations (p-values calculated using a two-tailed Mann–Whitney *U*-test). **e**, The proportion of ecDNA with kyklonas that contain multiple kyklonic events. The total number of ecDNA with kyklonas are displayed above each bar plot for each cancer type.

**Extended Data Fig. 9:**
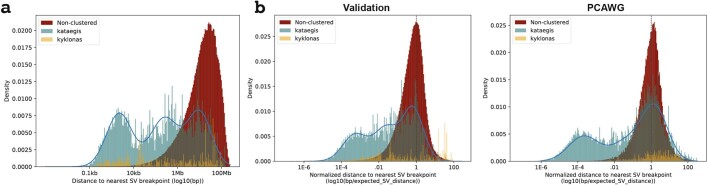
Kyklonas occur distally from structural breakpoints across three independent cohorts. **a**, The distance to the nearest breakpoint for all kataegic mutations (teal), kyklonas (gold), and non-clustered mutations (red) across the three validation cohorts. **b**, Distances to the nearest SV breakpoints were normalized by calculating the expected distance a mutation would fall from a breakpoint given the number of breakpoints detected per chromosome and the overall length of the chromosome across the validation cohorts (left) and PCAWG (right). A value of 1 (dashed line) reflects a distance that one would expect based on the random placement of a mutation across the chromosome, whereas a value less than 1 reflects a mutation occurring closer than what is expected by random chance. The distributions of kataegic mutations were modelled using Gaussian mixture models (blue lines) with an automatic selection criterion for the number of components using the minimum Bayesian information criteria (BIC).

**Extended Data Fig. 10:**
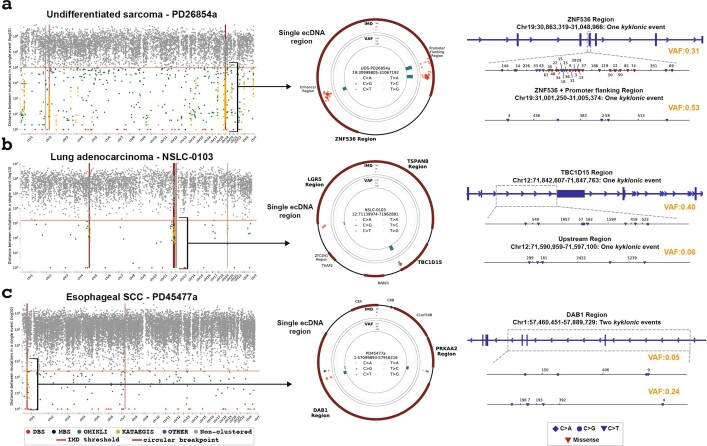
Examples of kyklonas in three independent cohorts. **a**, A single undifferentiated sarcoma genome depicting the overlap of kataegis with ecDNA regions displayed as a rainfall (left) with a single zoomed in ecDNA represented using a circos plot (middle). The outer track of the circos plot represents the reference genome of the ecDNA with proximal known cancer driver genes. The middle track reflects a circular rainfall plot where each dot represents the IMD around a single mutation coloured based on the substitution change. The innermost track shows the average VAF for each kyklonic event. Right: Two smaller regions of the selected ecDNA including a single kyklonic event within *ZNF536* region resulting in a plethora of missense and stop-gained mutations, and a single kyklonic event within a promoter flanking with the average VAFs per event (orange). **b**, A single lung adenocarcinoma genome depicting the overlap of kataegis with ecDNA regions (left) with a single zoomed in ecDNA containing *TBC1D15* and two distinct kyklonic events represented using a circos plot (middle). Right: Two kyklonic events overlapping an upstream region and *TBC1D15*. **c**, A single oesophageal squamous cell carcinoma genome depicting the overlap of kataegis with ecDNA regions (left) with a single zoomed in ecDNA containing *PRKAA2* and *DAB1* and three distinct kyklonic events (middle). Right: Two kyklonic events overlapping *DAB1*.

Kyklonic events were further investigated across 3 additional independent cohorts, including 61 sarcomas^44^, 280 lung cancers^45^ and 186 oesophageal squamous cell carcinomas^46^. Comparable rates of clustered mutagenesis were found for both substitutions and indels to the rates reported in PCAWG, with a 2.4- and 5.0-fold enrichment of clustered substitutions and indels within driver events, respectively (Extended Data Fig. 8a). Across the three cohorts, 31% of samples with ecDNA exhibited kyklonas within the sarcomas, 14% within the oesophageal cancers and 28% within the lung cancers, supporting the rates observed in PCAWG (Fig. 4c, Extended Data Figs. 7b, 8c). Similar to the rate observed in PCAWG (36.2%), approximately 30.1% of all kataegis occurred within 10 kb of the nearest breakpoint in the validation cohort (Extended Data Fig. 9a). In addition, only 0.34% of kyklonic events in the validation dataset occurred closer to SVs than expected by chance, which closely resembles the observations in the PCAWG data (0.35%) (Extended Data Fig. 9b). Kyklonic mutations were predominantly attributed to APOBEC3 signatures SBS2 and SBS13 (*P* < 1 × 10^−5^) (Extended Data Fig. 8b, Methods) with an enrichment of mutations at the RTCA context supporting the role of APOBEC3B (Extended Data Fig. 8d). A widespread recurrence of kyklonic events was observed across the sarcomas, oesophageal and lung cancers, with 45%, 28% and 46% of samples with ecDNA containing multiple, distinct kyklonic events (Extended Data Fig. 8e). An example from each cohort was selected to illustrate multiple kyklonic events occurring within single ecDNAs, validating the recurrent APOBEC3 hypermutation of ecDNA (Extended Data Fig. 10).

## Discussion

Clustered mutagenesis in cancer can occur through different mutational processes, with AID and APOBEC3 deaminases having the most prominent role. In addition to enzymatic deamination, other endogenous and exogenous sources imprint many of the observed clustered indels and substitutions. A multitude of mutational processes can give rise to omikli events, including tobacco carcinogens and exposure to ultraviolet light. Clustered substitutions and indels were highly enriched in driver events and associated with differential gene expression, implicating them in cancer development and cancer evolution. Some clustered mutational signatures are associated with known cancer risk factors or the activity or failure of DNA repair processes. Notably, clustered mutations in *TP53*, *EGFR* and *BRAF* associated with changes in overall survival and can be detected in most types of sequencing data, including clinically actionable targeted panels such as MSK-IMPACT.

A large proportion of kataegic events occur within 10 kb of detected SV breakpoints with a mutational pattern, suggesting the activity of APOBEC3. Multiple distinct kataegic events, independent of detected breakpoints, were observed on circular ecDNA; such events—termed kyklonas—suggest recurrent APOBEC3 mutagenesis. The circular topology of ecDNAs^47^ and their rapid replication patterns are reminiscent of the structure and behaviour of the circular genomes of several double-stranded-DNA based, pathogens including herpesviruses, papillomaviruses and polyomaviruses^32–35^. Previous pan-virome studies have shown that these double-stranded DNA viral genomes often manifest mutations from APOBEC3 enzymes^48–50^. As such, recurrent APOBEC3 mutagenesis on ecDNA is likely to be representative of an antiviral response in which the ecDNA viral-like structure is treated as an infectious agent and attacked by APOBEC3 enzymes. ecDNAs contain a plethora of cancer-associated genes and are responsible for many gene amplification events that can accelerate tumour evolution. Repeated mutagenic attacks of these ecDNAs reveal functional effects within known oncogenes and implicate additional modes of oncogenesis that may ultimately contribute to subclonal tumour evolution, subsequent evasion of therapy and clinical outcome. Further investigations with large-scale clinically annotated whole-genome-sequenced cancers are required to fully understand the clinical implications of clustered mutations and kyklonas.

## Methods

### Data sources

Somatic variant calls of single-base substitutions, small indels and structural variations were downloaded for the 2,583 white-listed whole-genome-sequenced samples from PCAWG along with the corresponding list of consensus driver events^10^. Epidemiological and clinical features for all available samples were downloaded from the official PCAWG release (https://dcc.icgc.org/releases/PCAWG). The collection of whole-exome-sequenced samples from TCGA along with all available clinical features were downloaded from the Genomic Data Commons (GDC; https://gdc.cancer.gov/). The MSK-IMPACT Clinical Sequencing Cohort^43^ composed of 10,000 clinical cases was downloaded from cBioPortal (https://www.cbioportal.org/study/summary?id=msk_impact_2017). The subclassification of focal amplifications comprised circular ecDNA, linear amplifications, BFBs and heavily rearranged events, and their corresponding genomic locations were obtained for a subset of samples (*n* = 1,291) as reported^34^.

Experimental models used to validate clustered events were derived from previous studies using primary Hupki mouse embryonic fibroblasts (MEFs) exposed to ultraviolet light^41^, human induced pluripotent stem cells (iPS cells) exposed to benzo[*a*]pyrene^40^, and a clonally expanded BT-474 human breast cancer cell line with episodically active APOBEC3^39^.

Independent cohorts used to validate kyklonic events were collected from multiple sources. The 61 undifferentiated sarcomas^44^ and 187 high-confidence oesophageal squamous cell carcinomas^46^ were downloaded from the European Genome-phenome Archive (EGAD00001004162 and EGAD00001006868, respectively). The 280 lung adenocarcinomas^45^ were downloaded from dbGaP under the accession number (phs001697.v1.p1). Clustered mutations in validation samples were analysed using the same approach as the one used in the original cohort.

### Detection of clustered events

SigProfilerSimulator (v.1.0.2) was used to derive an IMD cut-off^51^ that is unlikely to occur by chance based on the TMB and the mutational patterns for a given sample. Specifically, each tumour sample was simulated while maintaining the sample’s mutational burden on each chromosome, the ±2 bp sequence context for each mutation and the transcriptional strand bias ratios across all mutations. All mutations in each sample were simulated 100 times and the IMD cut-off was calculated such that 90% of the mutations below this cut-off could not appear by chance (*q*-value < 0.01). For example, in a sample with an IMD threshold of 500bp, one may observe 1,000 mutations within this threshold with no more than 100 mutations expected based on the simulated data (*q*-value < 0.01). *P* values were calculated using *z*-tests by comparing the number of real mutations and the distribution of simulated mutations that occur below the same IMD threshold. A maximum cut-off of 10 kb was used for all IMD thresholds. By generating a background distribution that reflects the random distribution of events used to reduce the false positive rate, this model also considers regional heterogeneities of mutation rates, partially attributed to replication timing and expression, and variances in clonality by correcting for mutation-rich regions and mutation-poor regions within 1-Mb windows. The 1-Mb window size has been used and established as an appropriate scale when considering the variability in mutation rates associated with chromatin structure, replication timing and genome architecture^14,52,53^. The 1-Mb window ensures that subsequent mutations are likely to have occurred as single events using a maximum cut-off of 0.10 for differences in the VAFs. The regional IMD cut-off was determined using a sliding window approach that calculated the fold enrichment between the real and simulated mutation densities within 1-Mb windows across the genome. The IMD cut-offs were further increased, for regions that had higher than ninefold enrichments of clustered mutations and where more than 90% of the clustered mutations were found within the original data, to capture additional clustered events while maintaining the original criteria (less than 10% of the mutations below this cut-off appear by chance; *q*-value < 0.01). Last, as VAF of mutations may confound the definition of clustered events in ecDNA, we calculated the distribution of inter-event distances within recurrently mutated ecDNA while disregarding the VAF of individual mutations. This resulted in the exact same separation of kataegic events using only the inter-event distances as a criterion for the grouping of mutations into a single event.

Subsequently, all clustered mutations with consistent VAFs were classified into one of four categories (Extended Data Fig. 1a). Two adjacent mutations with an IMD of 1 were classified as DBSs. Three or more adjacent mutations each with an IMD of 1 were classified as MBSs. Two or three mutations with IMDs less than the sample-dependent threshold and with at least a single IMD greater than 1 were classified as omikli. Four or more mutations with IMDs less than the sample-dependent threshold and with at least a single IMD greater than 1 were classified as kataegis. A cut-off of four mutations for kataegis was chosen by fitting a Poisson mixture model to the number of mutations involved in a single event across all extended clustered events excluding DBSs and MBSs (Supplementary Note 1). This model comprised two distributions with C1 = 2.08 and C2 = 4.37 representing omikli and kataegis, respectively. A cut-off of four mutations was used for kataegis on the basis of a contribution of greater than 95% from the kataegis-associated distribution with events of four or more mutations. Note that there is certain ambiguity for events with two or three mutations. Although the majority of these events are omikli, some of these events are likely to be short kataegic events (Supplementary Note 1). All remaining clustered mutations with inconsistent VAFs were classified as other. Clustered indels were not classified into different classes. We also performed additional quality-checks to ensure that the majority of clustered indels were mapped to high confidence regions of the genome (Supplementary Fig. 2). Specifically, all clustered indels were aligned against a consensus list of blacklisted genomic regions developed by ENCODE^54^ revealing that only 0.5% of all clustered indels overlapped regions with low mappability scores.

### Clustered mutational signatures analysis

The clustered mutational catalogues of the examined samples were summarized in SBS288 and ID83 matrices using SigProfilerMatrixGenerator^55^ (v.1.2.0) for each tissue type and each category of clustered events. For example, six matrices were constructed for clustered mutations found in Breast-AdenoCA: one matrix for DBSs, one matrix for MBSs, one matrix for omikli, one matrix for kataegis, one matrix for other clustered substitutions and one matrix for clustered indels. The SBS288 classification considers the 5′ and 3′ bases immediately flanking each single-base substitution (referred to using the pyrimidine base in the Watson–Crick base pair) resulting in 96 individual mutation channels. In addition, this classification considers the strand orientation for mutations that occur within genic regions resulting in three possible categories: (1) transcribed; pyrimidine base occurs on the template strand; (2) untranscribed; pyrimidine base occurs on the coding strand; or (3) non-transcribed; pyrimidine base occurs in an intergenic region. Mutations in genic regions that are bidirectionally transcribed were evenly split amongst the coding and template strand channels. Combined, this results in a classification consisting of 288 mutation channels, which were used as input for de novo signature extraction of clustered substitutions. The ID83 mutational classification has previously been described^55^.

Mutational signatures were extracted from the generated matrices using SigProfilerExtractor (v.1.1.0), a Python-based tool that uses non-negative matrix factorization to decipher both the number of operative processes within a given cohort and the relative activities of each process within each sample^56^. The algorithm was initialized using random initialization and by applying multiplicative updates using the Kullback–Leibler divergence with 500 replicates. Each de novo extracted mutational signature was subsequently decomposed into the COSMIC (v.3) set of signatures (https://cancer.sanger.ac.uk/signatures/) requiring a minimum cosine similarity of 0.80 for all reconstructed signatures. All de novo extractions and subsequent decomposition were visually inspected and, as previously done^1^, manual corrections were performed for 2.2% of extractions (4 out of 180 extractions) in which the total number of operative signatures was adjusted ±1. Consistent with prior visualizations^10^, we have included all cancer types within the PCAWG cohort, which may comprise as few as one sample for certain cancer types. Similarly, consistent with prior visualizations^1^, decomposed signature activity plots required that each cancer type have more than 2 samples and used mutation thresholds for each clustered category; 25 mutations per sample were required for DBSs, omikli events and other clustered mutations; 15 mutations per sample were required for MBSs and kataegic events; and 10 mutations were required per sample for clustered indels.

### Experimental validation

A subset of clustered mutational signatures was validated using previously sequenced in vitro cell line models. As done for PCAWG samples, we generated a background model using SigProfilerSimulator^51^ to calculate the clustered IMD cut-off for each sample and partitioned each substitution into the appropriate category of clustered events. Mutational spectra were generated for each subclass within each sample using SigProfilerMatrixGenerator^55^ and were compared against the de novo signatures extracted from human cancer. The cosine similarity between the in vitro mutational spectra and de novo observed clustered signatures was calculated to assess the degree of similarity. The average cosine similarity between two random non-negative vectors is 0.75, and the cosine similarities above 0.81 reflect *P* values below 0.01 (ref. ^51^).

### Associations with cancer risk factors

Homologous recombination (HR) deficiency was defined for breast cancers using the status of *BRCA1*, *BRCA2*, *RAD51C* and *PALB2*^57^. Samples with a germline, somatic or epigenetic alteration in one of these genes were considered HR-deficient, whereas samples without any known alterations in these genes were considered HR-proficient. The number of clustered indels was compared between HR-deficient and HR-proficient samples. The smoking status of lung cancers was determined using the clinical annotation from TCGA (https://portal.gdc.cancer.gov/repository). The number of clustered indels associated with tobacco smoking (ID6) was compared between samples annotated as lifelong non-smokers and samples annotated as current and reformed smokers. The status of alcohol consumption was determined using the annotations from the official PCAWG release (https://dcc.icgc.org/releases/PCAWG). The total number of clustered indels was compared in samples annotated with no alcohol consumption and those annotated as daily and weekly drinkers.

### Expression of driver genes

All RNA-seq expression data were downloaded as a part of the official PCAWG release (https://dcc.icgc.org/releases/PCAWG). The relative expression data found within this release were normalized using FPKM normalization and upper quartile normalization. The relative expression of a gene was compared between those containing clustered or non-clustered events. Each distribution was then normalized to the average expression of the wild-type gene. Only genes with at least 10 total events (that is, clustered and non-clustered mutations) including at least 5 clustered events were considered for examination.

### SVs and clustered events

The distance to the nearest structural variation breakpoint was calculated for each mutation in each subclass using the minimum distance to the nearest adjacent upstream or downstream breakpoint. Each distribution was modelled using a Gaussian mixture with an automatic selection criterion for the number of components ranging between one and five components using the minimum Bayesian information criteria (BIC) across all iterations. Modelling of kataegic events resulted in an optimal fit of three components, which was used to separate kataegic substitutions into SV-associated and non-SV associated mutations. DBSs and MBSs were both modelled using a single Gaussian distribution relating to non-SV associated mutations, whereas omikli and other clustered mutations were modelled using a mixture of two components, probably reflecting leakage of smaller kataegic events contributing to a weak SV-associated distribution. To account for the frequency of breakpoints across each sample, we normalized the minimum distance of each mutation to the nearest SV by calculating the expected distance between a mutation and SV for each sample using the total number of breakpoints and the overall length of a given chromosome (Extended Data Fig. 9a, b). After normalizing the kataegic events, we observed an optimal solution of two components with one SV-associated distribution (on average each mutation occurs within one-thousandth of the expected distance to nearest structural variation) and one non-SV associated distribution (on average occurring within the expected distance to the nearest structural variation). The normalized kyklonic events are consistent with the non-SV associated distribution reflecting kataegic events that occur on ecDNA typically of lengths 1–10 Mb (ref. ^35^).

### APOBEC3A and APOBEC3B enrichment analysis

The enrichment score of RTCA and YTCA penta-nucleotides quantifies the frequency for which each TpCpA>TpKpA mutation occurs at either an RTCA or a YTCA context. To account for motif availability, this score is calculated using the ±20 bp sequence context around each mutation and normalized by the number of cytosine bases and C>N mutations within the set of 41-mers surrounding each mutation of interest^7^.

### APOBEC3 gene expression and kyklonas

All RNA-seq expression data were downloaded as a part of the official PCAWG release (https://dcc.icgc.org/releases/PCAWG). The relative expression data found within this release were normalized using FPKM normalization and upper quartile normalization. The APOBEC3A/B normalized expression was compared between samples containing ecDNA versus samples with no detected ecDNA and between samples with kyklonas and without kyklonas. All *P* values were generated using a Mann–Whitney *U*-test and were corrected for multiple hypothesis testing using the Benjamini–Hochberg FDR procedure.

### Circular ecDNA and kataegis

The collection of ecDNA ranges was intersected with the catalogue of clustered mutations, which was used to determine the overlapped mutational burden for each subclass of clustered event and the mutational spectra of overlapping kataegic events. Enrichments of events were calculated using statistical background models generated using SigProfilerSimulator^51^ that shuffled the dominant mutation in each clustered event across the genome (that is, the most frequent mutation type in a single event). The decomposed kyklonic mutational spectra were generated using the decomposition module within SigProfilerExtractor^56^. Only mutational signatures that increased the overall cosine similarity by at least 0.01 were used. In both the original and validation cohorts, SBS2 and SBS13 were sufficient to explain the kyklonic mutational spectra with no other known mutational signature increasing the cosine similarity by more than 0.01. Comparisons between ecDNA with and without cancer genes were performed using the set of cancer genes from the Cancer Gene Census (CGC)^58^. All statistical comparisons and *P* values were calculated using a two-tailed Mann–Whitney *U*-test unless otherwise specified. For each set of tests, *P* values were corrected for multiple hypothesis testing using the Benjamini–Hochberg FDR procedure. The predicted effect of each overlapping variant was determined using ENSEMBL’s Variant Effect Predictor tool by reporting only the most severe consequence^59^.

### Overall survival and clustered mutations

All survival analyses, including the generation of Kaplan–Meier curves, Cox regressions and log-rank tests, were performed using the Lifelines Python package (v.0.24.4). Across the 30 distinct whole-genome-sequenced cancer types included in the PCAWG study, only 6 cancer types contained enough samples to examine the associations between survival and overall number of clustered mutations. The sufficient sample size criteria required more than 50 samples with survival end-points with at least 30 of the samples with an observed clustered event. Each cancer type was analysed separately by comparing the survival of samples with a high clustered mutational burden (top 80th percentile across a given cancer type) to the survival of samples with a low clustered mutational burden (bottom 20th percentile across a given cancer type).

Analysis of whole-exome-sequenced samples from TCGA was altered to reflect the limited resolution for identifying clustered mutations within the exome. Specifically, SigProfilerSimulator (v.1.0.2)^51^ was used to derive an IMD cut-off for each sample based on the TMB within the exome and the mutational patterns for a given sample. Mutations were randomly shuffled while maintaining the mutational burden within the exome of each chromosome, the ±2 bp sequence context for each mutation and the transcriptional strand bias ratios across all mutations. Each sample was simulated 100 times and an IMD cut-off was calculated using the same methods as outlined for the detection of clustered events within PCAWG. Owing to the limited number of detected events, 22 cancer types had sufficient data to perform survival analysis. Each cancer type was analysed separately by comparing samples with at least a single clustered event to samples with no detected clustered events within the exome.

For both PCAWG and TCGA analyses, survival distributions within a given cancer type were compared using a log-rank test. Cox regressions were performed to determine hazards ratios and to correct for age and total mutational burden. All *P* values were also corrected for multiple hypothesis testing using the Benjamini–Hochberg FDR procedure.

To investigate differential survival associated with the detection of clustered events within cancer driver genes, Kaplan–Meier survival curves were compared between individuals with clustered versus non-clustered mutations within a given cancer driver gene. The distributions were compared using a log-rank test. Cox regressions were performed to determine the hazards ratios and to correct for age, total mutational burden and cancer type across TCGA. Cox regressions performed for the MSK-IMPACT cohort were corrected for total mutational burden and cancer type. No corrections were performed for age as these metadata were not available for the MSK-IMPACT cohort. All *P* values were also corrected for multiple hypothesis testing using the Benjamini–Hochberg FDR procedure.

### Validation of kyklonas in three cohorts

All three validation cohorts were analysed analogous to the PCAWG cohorts. Specifically, clustered mutations were classified by calculating a sample-dependent IMD threshold for clustered versus non-clustered mutations using a background model generated by SigProfilerSimulator^51^. All clustered mutations were subclassified into DBS, MBS, omikli, kataegis or other mutations. AmpliconArchitect (v.1.2) was used to determine regions of focal amplifications^60^, which were used for subsequent validation of kyklonic events by overlapping kataegic events with all detected focal amplifications. The decomposed kyklonic mutational spectra were generated using the decomposition module within SigProfilerExtractor^56^. Only mutational signatures that increased the overall cosine similarity by at least 0.01 were used. In both the original and validation cohorts, SBS2 and SBS13 were sufficient to explain the kyklonic mutational spectra with no other known mutational signature increasing the cosine similarity by more than 0.01.

### Cancer-type abbreviations

Biliary-AdenoCA, biliary adenocarcinoma; Bladder-TCC, bladder transitional cell carcinoma; Bone-Epith, bone epithelioid; Bone-Leiomyo, bone leiomyosarcoma; Bone-Osteosarc, bone osteosarcoma; Breast-AdenoCA, breast adenocarcinoma; Breast-LobularCA, breast lobular carcinoma; CNS-GBM, glioblastoma (central nervous system); CNS-Medullo, medulloblastoma (central nervous system); CNS-Oligo, oligodendroglioma (central nervous system); CNS-PiloAstro, pilocytic astrocytoma (central nervous system); Cervix-AdenoCA, cervix adenocarcinoma; Cervix-SCC, cervix squamous cell carcinoma; ColoRect-AdenoCA, colorectal adenocarcinoma; Head-SCC, head and neck squamous cell carcinoma; Kidney-ChRCC, chromophobe renal cell carcinoma; Kidney-RCC, renal cell carcinoma; Liver-HCC, hepatocellular carcinoma; Lung-AdenoCA, lung adenocarcinoma; Lung-SCC, lung squamous cell carcinoma; Lymph-BNHL, B-cell non-Hodgkin lymphoma; Lymph-CLL, chronic lymphocytic leukaemia; Lymph-NOS, metastatic lymphoma; Myeloid-AML, acute myeloid leukaemia; Myeloid-MPN, myeloproliferative neoplasm; Oeso-AdenoCA, oesophageal adenocarcinoma; Ovary-AdenoCA, ovary adenocarcinoma; Panc-AdenoCA, pancreatic adenocarcinoma; Panc-Endocrine, pancreatic neuroendocrine carcinoma; Prost-AdenoCA, prostate adenocarcinoma; Skin-Melanoma, malignant melanoma; Stomach-AdenoCA, stomach adenocarcinoma; Thy-AdenoCA, thyroid adenocarcinoma; Uterus-AdenoCA, uterine adenocarcinoma.

### Reporting summary

Further information on research design is available in the Nature Research Reporting Summary linked to this paper.

## Online content

Any methods, additional references, Nature Research reporting summaries, source data, extended data, supplementary information, acknowledgements, peer review information; details of author contributions and competing interests; and statements of data and code availability are available at 10.1038/s41586-022-04398-6.

## Supplementary information

Supplementary Information This file contains Supplementary Note 1; Supplementary Figures 1 and 2 and Supplementary References

Reporting Summary

Supplementary Table 1 Mutational signature activities of double-base substitutions

Supplementary Table 2 Mutational signature activities of multi-base substitutions

Supplementary Table 3 Mutational signature activities of omikli events

Supplementary Table 4 Mutational signature activities of kataegic events

Supplementary Table 5 Mutational signature activities of clustered indel events

Supplementary Table 6 Mutational signature activities of other events

Supplementary Table 7 Coding kyklonic mutations within ecDNA

## Data Availability

No data were generated specifically for this study. All data were and can be downloaded from the appropriate links, repositories and references. Specifically, for the discovery cohort, all data and metadata were obtained from the official PCAWG release (https://dcc.icgc.org/releases/PCAWG). All data and metadata for TCGA samples were obtained from the GDC (https://gdc.cancer.gov/). Genomics data for clonally expanded cell lines were downloaded from the European Genome-phenome Archive (EGAD00001004201, EGAD00001004203 and EGAD00001004583). For the three validation cohorts, datasets were downloaded as submitted by the original publications and genomics data were downloaded from their respective repositories: EGAD00001004162 for 61 undifferentiated sarcomas^44^ (European Genome-phenome Archive); EGAD00001006868 for 187 high-confidence oesophageal squamous cell carcinomas^46^ (European Genome-phenome Archive); and phs001697.v1.p1 for 280 lung adenocarcinomas^45^ (dbGaP). Somatic mutations and metadata for the MSK-IMPACT Clinical Sequencing Cohort composed of 10,000 clinical cases^42^ were downloaded from cBioPortal (https://www.cbioportal.org/study/summary?id=msk_impact_2017).

## Code availability

The SigProfiler compendium of tools are developed as Python packages and are freely available for installation through PyPI or directly through GitHub (https://github.com/AlexandrovLab/). For all tools, each package is fully functional, free and open sourced distributed under the permissive 2-Clause BSD License and is accompanied by extensive documentation: (1) SigProfilerMatrixGenerator^55^ (v.1.2.0; https://github.com/AlexandrovLab/SigProfilerMatrixGenerator); (2) SigProfilerSimulator^51^ (v.1.0.2; https://github.com/AlexandrovLab/SigProfilerSimulator); and (3) SigProfilerExtractor^56^ (v.1.1.0; https://github.com/AlexandrovLab/SigProfilerExtractor). Each SigProfiler tool also has an R wrapper available for installation through the GitHub repositories. AmpliconArchitect^34^ (v.1.2) is also freely available and can downloaded from https://github.com/virajbdeshpande/AmpliconArchitect. The core computational pipelines used by the PCAWG Consortium for alignment, quality control and variant calling are available to the public at https://dockstore.org/search?search=pcawg under the GNU General Public License v.3.0, which allows for reuse and distribution.
