# Supplementary material for: Mapping clustered mutations in cancer reveals APOBEC3 mutagenesis of ecDNA
Source: Nature. 2022 Feb 9;602(7897):510–7. doi: 10.1038/s41586-022-04398-6 (PMC8850194; doi:10.1038/s41586-022-04398-6)
Supplement: Supplementary file 1 — This file contains Supplementary Note 1; Supplementary Figures 1 and 2 and Supplementary References [file 41586_2022_4398_MOESM1_ESM.pdf]

nature portfolio

https://doi.org/10.1038/s41586-022-04398-6

Supplementary information

Mapping clustered mutations in cancer reveals APOBEC3 mutagenesis of ecDNA

In the format provided by the authors and unedited

Supplementary Note 1 for *Mapping clustered mutations in cancer reveals APOBEC3 mutagenesis of ecDNA*

Erik N. Bergstrom, Jens Luebeck, Mia Petljak, Azhar Khandekar, Mark Barnes, Tongwu Zhang, Christopher D Steele, Nischalan Pillay, Maria Teresa Landi, Vineet Bafna, Paul S. Mischel, Reuben S. Harris, Ludmil B. Alexandrov

Table of Contents

Supplementary Note 1. Additional technical analysis of clustered mutagenesis. 2

1. Supplementary Figure 1. Determining the number of mutations differentiating between *omikli* and *kataegis*. 2

2. Supplementary Figure 2. The distribution of low confidence clustered indels. 3

Supplementary References 4

Supplementary Note 1. Additional technical analysis of clustered mutagenesis. The note contains technical and methodological information about differentiating between omikli and kataegis as well as about examining low confidence clustered indels.

1. Determining the number of clustered mutations in *omikli* and *kataegic* events.

To determine the cutoff of the number of mutations in an *omikli* versus a *kataegic* event, we modelled the distribution of clustered event sizes (excluding DBSs, MBSs, and *other* clustered events with disagreeable variant allele frequencies) using a mixture of two Poisson distributions (**Supplementary Figure 1*a***). The modelling also excluded clustered mutations from skin melanomas, that contribute a disproportionate number of DBS events, and clustered mutations from lymphomas, that contribute a large proportion of canonical and non-canonical AID *kataegis*. The first component, corresponding with *omikli* events (gold), had an average of 2.1 mutations per event, while the second component, corresponding to larger *kataegic* events (teal), had an average of 4.4 mutations per events. Using the posterior probabilities of each distribution, we calculated the likelihood of a given clustered event belonging to a specific component. Events comprised of four or more mutations were attributed to the *kataegic* component with >95% probability. Further, we assessed the IMD distributions of different sized events revealing approximately a 2-fold increase in average IMD between events possessing 3 and 4 mutations supporting the activity of two separate mutational processes (**Supplementary Figure 1*b***).

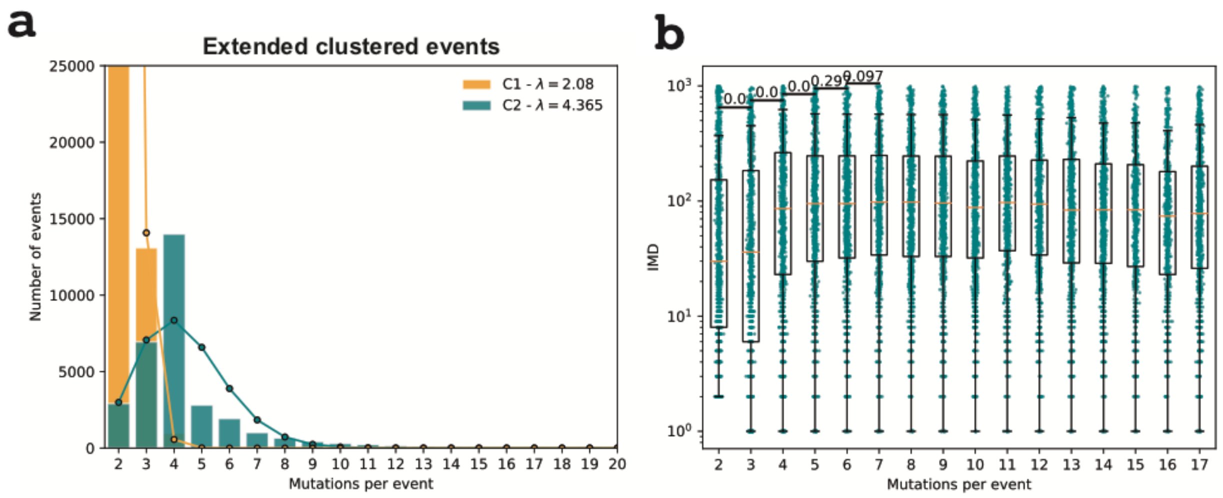

Supplementary Figure 1: Determining the number of mutations differentiating between *omikli* and *kataegis*. *a)* Modeling the number of mutations per event using a mixture of two Poisson distributions. The first component, representative of *omikli*, has an average IMD of 2.1, while the second component, representative of *kataegis*, has an average IMD of 4.4. The estimated contribution of mutations of each component are depicted as bars for each corresponding event size ***b)*** The distribution of IMDs per event across different sized events (n=199,912 events with 2 mutations; n=35,576 events with 3 mutations; n=15,320 events with 4 mutations; n=9,613 events with 5 mutations). The chosen cutoff between *omikli* and *kataegis* was four mutations. For each boxplot, the middle line reflects the median, the lower and upper bounds of the box correspond to the first and third quartiles, and the lower and upper whiskers extend from the box by 1.5x the inter-quartile range (IQR).

2. Analyzing the mapping scores of clustered indels.

We examined the mapping scores across the genome for clustered indels to ensure that the majority of events fall within high confidence regions. For this analysis we used a consensus list of blacklisted genomic regions developed by ENCODE[1] and the complete set of clustered indels as identified from the 2,583 PCAWG samples.

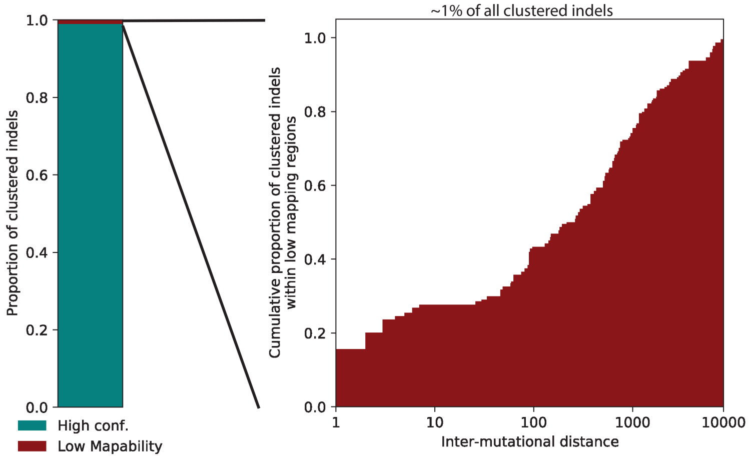

Supplementary Figure 2. The distribution of low confidence clustered indels. The number of clustered indels falling within regions of the genome with low mapping scores consists of approximately 1% of all clustered indels. Within these 1% of mutations with low mapping scores, only 30% of events have an inter-mutational distance less than 10 (0.3% of all clustered indels), while indels of lbp falling within low mapping regions comprise only 0.5% of all clustered indels.

Supplementary References

1 Amemiya, H. M., Kundaje, A. & Boyle, A. P. The ENCODE Blacklist: Identification of Problematic Regions of the Genome. *Sci Rep* **9**, 9354, doi:10.1038/s41598-019-45839-z (2019).
