# Supplementary material for: Mapping clustered mutations in cancer reveals APOBEC3 mutagenesis of ecDNA
Source: Nature. 2022 Feb 9;602(7897):510–7. doi: 10.1038/s41586-022-04398-6 (PMC8850194; doi:10.1038/s41586-022-04398-6)
Supplement: Supplementary file 2 — Reporting Summary [file 41586_2022_4398_MOESM2_ESM.pdf]

nature research

Corresponding author(s): Ludmil B. Alexandrov

Last updated by author(s): Dec 14, 2021

Reporting Summary

Nature Research wishes to improve the reproducibility of the work that we publish. This form provides structure for consistency and transparency in reporting. For further information on Nature Research policies, see our Editorial Policies and the Editorial Policy Checklist.

Statistics

For all statistical analyses, confirm that the following items are present in the figure legend, table legend, main text, or Methods section.

n/a	Confirmed	
☐	☒	The exact sample size (*n*) for each experimental group/condition, given as a discrete number and unit of measurement
☐	☒	A statement on whether measurements were taken from distinct samples or whether the same sample was measured repeatedly
☐	☒	The statistical test(s) used AND whether they are one- or two-sided<br>*Only common tests should be described solely by name; describe more complex techniques in the Methods section.*
☐	☒	A description of all covariates tested
☐	☒	A description of any assumptions or corrections, such as tests of normality and adjustment for multiple comparisons
☐	☒	A full description of the statistical parameters including central tendency (e.g. means) or other basic estimates (e.g. regression coefficient) AND variation (e.g. standard deviation) or associated estimates of uncertainty (e.g. confidence intervals)
☐	☒	For null hypothesis testing, the test statistic (e.g. *F*, *t*, *r*) with confidence intervals, effect sizes, degrees of freedom and *P* value noted<br>*Give P values as exact values whenever suitable.*
☒	☐	For Bayesian analysis, information on the choice of priors and Markov chain Monte Carlo settings
☒	☐	For hierarchical and complex designs, identification of the appropriate level for tests and full reporting of outcomes
☐	☒	Estimates of effect sizes (e.g. Cohen's *d*, Pearson's *r*), indicating how they were calculated

Our web collection on statistics for biologists contains articles on many of the points above.

Software and code

Policy information about availability of computer code

Data collection	No data were generated specifically for this study. All data were and can be downloaded from the appropriate links, repositories, and references. Specifically, for the discovery cohort, all data and metadata were obtained from the official PCAWG release: https://dcc.icgc.org/releases/PCAWG. All data and metadata for TCGA samples were obtained from GDC: https://gdc.cancer.gov/. Genomics data for clonally expanded cell lines were downloaded from European Genome-phenome Archive: EGAD00001004201, EGAD00001004203, and EGAD00001004583. For the three validation cohorts, datasets were downloaded as submitted by the original publications and genomics data were downloaded from their respective repositories: EGAD00001004162 for 61 undifferentiated sarcomas (European Genome-phenome Archive), EGAD00001006868 for 187 high-confidence esophageal squamous cell carcinomas (European Genome-phenome Archive), and phs001697.v1.p1 for 280 lung adenocarcinomas (dbGaP). Somatic mutations and metadata for the MSK-IMPACT Clinical Sequencing Cohort composed of 10,000 clinical cases were downloaded from cBioPortal: https://www.cbioportal.org/study/summary?id=msk_impact_2017.
Data analysis	The SigProfiler compendium of tools are developed as Python packages and are freely available for installation through PyPI or directly through GitHub (https://github.com/AlexandrovLab/). For all tools, each package is fully functional, free, and open sourced distributed under the permissive 2-Clause BSD License and are accompanied by extensive documentation: (i) SigProfilerMatrixGenerator (version 1.2.0; https://github.com/AlexandrovLab/SigProfilerMatrixGenerator); (ii) SigProfilerSimulator: (version 1.0.2; https://github.com/AlexandrovLab/SigProfilerSimulator); (iii) SigProfilerExtractor: (version 1.1.0; https://github.com/AlexandrovLab/SigProfilerExtractor). Each SigProfiler tool also has an R wrapper available for installation through the GitHub repositories. AmpliconArchitect (version 1.2) is also freely available and can downloaded from https://github.com/virajbdeshpande/AmpliconArchitect. The core computational pipelines used by the PCAWG Consortium for alignment, quality control and variant calling are available to the public at https://dockstore.org/search?search=pcawg under the GNU General Public License v.3.0, which allows for reuse and distribution.

For manuscripts utilizing custom algorithms or software that are central to the research but not yet described in published literature, software must be made available to editors and reviewers. We strongly encourage code deposition in a community repository (e.g. GitHub). See the Nature Research guidelines for submitting code & software for further information.

Data

Policy information about availability of data

All manuscripts must include a data availability statement. This statement should provide the following information, where applicable:

- Accession codes, unique identifiers, or web links for publicly available datasets
- A list of figures that have associated raw data
- A description of any restrictions on data availability

No data were generated specifically for this study. All data were and can be downloaded from the appropriate links, repositories, and references. Specifically, for the discovery cohort, all data and metadata were obtained from the official PCAWG release: https://dcc.icgc.org/releases/PCAWG. All data and metadata for TCGA samples were obtained from GDC: https://gdc.cancer.gov/. Genomics data for clonally expanded cell lines were downloaded from European Genome-phenome Archive: EGAD00001004201, EGAD00001004203, and EGAD00001004583. For the three validation cohorts, datasets were downloaded as submitted by the original publications and genomics data were downloaded from their respective repositories: EGAD00001004162 for 61 undifferentiated sarcomas (European Genome-phenome Archive), EGAD00001006868 for 187 high-confidence esophageal squamous cell carcinomas (European Genome-phenome Archive), and phs001697.v1.p1 for 280 lung adenocarcinomas (dbGaP). Somatic mutations and metadata for the MSK-IMPACT Clinical Sequencing Cohort composed of 10,000 clinical cases were downloaded from cBioPortal: https://www.cbioportal.org/study/summary?id=msk_impact_2017.

Field-specific reporting

Please select the one below that is the best fit for your research. If you are not sure, read the appropriate sections before making your selection.

☒ Life sciences ☐ Behavioural & social sciences ☐ Ecological, evolutionary & environmental sciences

For a reference copy of the document with all sections, see nature.com/documents/nr-reporting-summary-flat.pdf

Life sciences study design

All studies must disclose on these points even when the disclosure is negative.

Sample size	No sample size calculation was performed as no data were being generated. Rather, all publicly available samples were utilized in our analysis and, for each comparison sufficient numbers were determined based on a FDR-corrected statistically significant p-value and magnitude of effect size. Overall, the study utilized the complete set of 2,583 white-listed whole-genome sequenced samples from PCAWG along with their corresponding list of consensus driver events. Samples were taken as provided by the PCAWG consortium. Extrachromosomal-DNA (ecDNA) can be unambiguously assigned to 1,291 of these samples. Validation cohorts included 61 sarcomas, 280 lung cancers, and 186 esophageal squamous cell carcinomas.
Data exclusions	No samples were exclude in the discovery PCAWG cohort. All PCAWG cancer types with more than 10 samples are presented within the main figures while cancer types with less than 10 samples are included in Extended Data Figures. In the validation esophageal cohort, only high-confidence esophageal squamous cell carcinomas were used as annotated in the submission to the data repository. No samples were excluded from the sarcoma and lung cancer validation cohorts.
Replication	Replication of genomics analyses encompassed three independent cohorts and a total of 527 additional whole-genome sequenced samples, including: 61 sarcomas, 280 lung cancers, and 186 esophageal squamous cell carcinomas. The results from the genomics analyses of PCAWG were replicated three times -- one per each validation cohort. Additionally, the MSK-IMPACT Clinical Sequencing Cohort composed of 10,000 clinical cases was used for clinical validation. The results from the clinical analysis of TCGA clustered cancer genes was replicated one time -- in the MSK-IMPACT Clinical Sequencing Cohort.
Randomization	There was no sample randomization in this study. Rather, the performed statistical analyses controlled for most known confounders. Specifically, in the clinical association analyses, we corrected for age of diagnosis (where available), tumor mutational burden, and cancer type. For most statistical comparisons between clustered mutations, a correction was performed based on observed behavior of non-clustered mutations.
Blinding	Detection of clustered mutations was performed independently and in a blinded manner in regard to driver mutations, overall survival, and identification of extrachromosomal-DNA (ecDNA).

Reporting for specific materials, systems and methods

We require information from authors about some types of materials, experimental systems and methods used in many studies. Here, indicate whether each material, system or method listed is relevant to your study. If you are not sure if a list item applies to your research, read the appropriate section before selecting a response.

Materials & experimental systems

n/a	Involved in the study
☒	☐ Antibodies
☒	☐ Eukaryotic cell lines
☒	☐ Palaeontology and archaeology
☒	☐ Animals and other organisms
☒	☐ Human research participants
☒	☐ Clinical data
☒	☐ Dual use research of concern

Methods

n/a	Involved in the study
☒	☐ ChIP-seq
☒	☐ Flow cytometry
☒	☐ MRI-based neuroimaging
